# Occurrence of mammary gland tumours in male dogs and its weak association with development of testicular tumours: a review

**DOI:** 10.1007/s13353-023-00818-z

**Published:** 2023-12-21

**Authors:** Angelika Tkaczyk-Wlizło, Krzysztof Kowal, Anna Śmiech, Brygida Ślaska

**Affiliations:** 1 Institute of Biological Bases of Animal Production, Faculty of Animal Sciences and Bioeconomy, University of Life Sciences in Lublin, 13 Akademicka St, 20-950 Lublin, Poland; 2 Department of Pathomorphology and Forensic Medicine, Faculty of Veterinary Medicine, University of Life Sciences in Lublin, 30 Głęboka St, 20-612 Lublin, Poland

**Keywords:** Cancer, Canine, MGT, Neoplasm, Testis

## Abstract

Mammary gland tumours (MGTs) are commonly occurring neoplasms in female dogs. However, rare cases of MGTs in male dogs have been reported for years. Due to the low incidence of MGTs in male dogs in comparison to female dogs, veterinary oncology is mainly focused on mammary neoplasms diagnosed in female dogs and extensive research is conducted in this scientific area. Therefore, there are no sufficient epidemiological data on male dogs and the aetiology of their tumour development is still poorly understood.

The aim of this literature review was to present cases of MGTs in male dogs for better understanding the scale of the problem over the years. The analyses of 74 affected male dogs with 92 tumours showed that the majority of MGTs in male dogs were benign tumours (54.3%), especially in form of adenomas, often developed in posterior canine mammary glands (58.1%).

The increased number of canine MGTs in male dogs aged 7 -13 years with an age peak at 11 years was noted. The age of affected animals was not related to breed. Mammary gland neoplasms were diagnosed predominately in Crossbreeds (20.2%) followed by Cocker Spaniels (18.9%) and German Shepherds (10.8%).

The association between MGT development in male dogs and co-occurrence of testicular tumours (TTs) has been discussed for years. Thus, cases of development of both tumours were included in this study. As a result, only in 12.7% cases of MGTs also history of TTs was described. Therefore, no general association between these tumours should be assumed.

## Introduction

Mammary gland tumours (MGTs) are commonly diagnosed neoplasms in female dogs representing from 25 to 50% off all tumours identified in bitches (Collivignarelli et al. 2021). Also, rare examples of MGTs are reported in male dogs. However, as emphasised by other authors, there are still insufficient data about MGTs in male dogs (Figueiroa et al. 2012; Maiti et al. 2014; Saba et al. 2007; Silva et al. 2019). A comparison of the occurrence of MGTs in male vs. female dogs shows a low incidence ranging from 1:50 (Mulligan 1949) to 1:62 (Saba et al. 2007), depending on studies.

The mammary gland is a dynamic organ present in all mammals, which is regulated by the coordinated action of reproductive and metabolic hormones. These hormones are responsible for promotion of gland development and its reconstruction to a milk-secreting organ during pregnancy (Schulman et al. 2022; Sorenmo et al. 2011).

However, sex hormones such as oestradiol also play an important role in mammary carcinogenesis. Currently, it is assumed that the molecular mechanisms of action of these molecules include the classic genomic effects modulating gene transcription and non-genomic effects, which trigger quick effects after a hormone binds to its specific receptors. These responses modulate various intracellular signalling pathways, triggering post-translational modification of several proteins (Torres et al. 2021).

The occurrence of MGTs in male dogs is definitely not as common as in bitches, but many cases of this disease have been reported over the years (Bearss et al. 2012; Han et al. 2016; Jabara 1969; Lather et al. 2017; Tkaczyk-Wlizło et al. 2023). Interestingly, in some cases of MGTs, also testicular tumours (TTs) and/or testicular abnormalities were observed (Kwon et al. 2017; Muscatello et al. 2021; Walker 1968; Warland et al. 2011; Zuchi et al. 2018). Since the male reproductive system is hormone-dependent, some authors suggest an association between the occurrence of MGTs and TTs (Maiti et al. 2014; Walker 1968).

Although the first case of MGT in a male dog was published as early as in 1936 (Jackson 1936), it seems that there are only a few other papers on this problem (Bearss et al. 2012; Maiti et al. 2014; Saba et al. 2007). Therefore, the aim of this paper was to present all available data of cases of MGTs in male dogs reported for years and to characterise the important individual properties as well as the histopathological and clinical features of male MGTs.

Moreover, some authors observed an association between some cases of MGTs and testicular neoplasms while others do not agree with this finding. Therefore, this review also collects cases of double-types of tumours (MGT, TT).

## Mammary gland development in dogs

The formation of the mammary glands takes place during the embryonic development when two ventral linear thickenings (ridges) of the ectoderm with specialised regions of the mesoderm below occur. Next, the ridges (milk lines) run in the anteroposterior direction from the fore- to the hindlimb. The ectodermal cells migrate along with each milk line and collocate to form a placode, which finally becomes an individual mammary gland (Silver 1966; Sorenmo et al. 2011).

The formation of the placode is a complex interaction involving many signal pathways between selected germ layers. As a result, a solid cord of epithelial cells is created and grows into the underlying mesenchyme to form mammary buds, which subsequently branch to form a mammary sprout. In female dogs, mammary sprouts undergo cavitation to form a lumen in each mammary sprout; thus, epithelial lined lactiferous ducts develop. Generally, the structure of the male mammary gland is similar to that in females, but the main difference is the lack of a system of branchy ducts and specialised lobules which develop during puberty and gestation, respectively (Silver 1966; Sorenmo et al. 2011). Each dog usually develops five symmetrically (left, right) located pairs of mammary glands including: cranial thoracic (1st), caudal thoracic (2nd), cranial abdominal (3rd), caudal abdominal (4th), and inguinal (5th) glands (Silver 1966).

## Mammary gland tumours in male dogs

MGTs are not commonly diagnosed neoplasms in male dogs, although many cases have been reported for years (Bearss et al. 2012; Han et al. 2016; Kwon et al. 2017). To date, some neoplasms such as complex adenoma (Han et al. 2016), fibroadenoma (Maiti et al. 2014), inflammatory carcinoma (Silva et al. 2019), simple adenoma (Bearss et al. 2012, Maiti et al. 2014), and simple carcinoma (Figueiroa et al. 2012) have been diagnosed in male MGTs.

As suggested by Silva et al. (2019), it is difficult to draw conclusions about the occurrence of selected types of MGTs from individual cases or from a limited number of affected male dogs. Therefore, for better understanding of some of the associations, each case of a male dog with MGT for which basic data such as the breed, age, sexual status, and tumour characteristics were available is presented in Table 1. It should be noted that a few cases of dogs: one benign mixed tumour, three adenocarcinomas, four malignant mixed tumours, one complex carcinoma, one simple adenoma, and three male dogs for which detailed characteristics was not available (Chae et al. 2007; Dhami et al. 2010; Mitchell et al. 1974; Patel et al. 2019; Sangha et al. 2012) are omitted in Table 1 because of the limited information.

**Table 1:** Occurrence of mammary gland tumours (MGTs) in male dogs including benign neoplasms, carcinomas, and hyperplasia/dysplasia and accompanying testicular tumours (TTs) and/ or cryptorchidism with indicated immunohistological markers

No	Breed of dog	Age [years]	Size of the dog ^a^	Sexual status	MGT localisation	MGT type ^b^	Number of MGT
1	Akita	12	M	n.d.^d^	A. 1st right B. 2nd right C. 3rd right	MC	3
2	American Pit Bull Terrier	11	L	intact	right posterior		1
3	American Staffordshire Terrier	11*	L	neutered	1st or 2nd left^e^	BN	
4	Basset Hound	7–13^ g^	M	n.d	n.d		
5	Boxer	7^ h^	L		4th right 5th right		2
6	Cocker Spaniel	4*	M	neutered	4th right 5th right		
7		7*		intact	left of prepuce^e^		1
8					A., B. left of prepuce C., D. right of prepuce		4
9				neutered	3rd left		1
10					2nd left		
11		12 * *		intact	left of prepuce^e^		2
12					3rd right		1
13				neutered	n.d		2
14		13		intact	A. 4th right, 1st right, B. 5th left	MC	2 1
15		14*		neutered	A. 1st left B. 2nd left C. 3rd left D. 1st right	BN	4
16		15	M	n.d	5th^e^	MC	1
17		7–13^ g^			n.d	BN	
18							
19						BN, MC, H/D	
20	Crossbreed	7	M	n.d	n.d	MC	1
21		9*	n.d	intact	4th left	BN	
22		10	M			MC	
23			n.d		5th right		
24		11* ^h^			4th left	BN	
25					4th right	MM	
26			M	n.d	5th left	MC	
27			L	intact	1st left	BN	
28		13		n.d		MC	
29			n.d	intact	n.d		
30							
31		14		n.d	4th^e^		
32	Crossbreed in Australian Kelpie type	14	M	n.d	5th^5^		
33	Crossbreed in Bullmastiff type	10*	L	neutered	level of prepuce ^e^	BN	
34	Crossbreed in Shih Tzu type	14*	S		n.d	H/D	
35	Dachshund	11*		neutered	level of prepuce ^e^	BN	
36	Doberman Pinscher	10*	L	intact			
37	Dogue de Bordeaux	7		n.d	level of prepuce (right side) ^e^		
38	Dogo Argentino	9			n.d	MC	many
39	English Bulldog	6*	S	neutered	1st or 2nd left^e^	BN	1
40		7–13^ g^		n.d	n.d		
41	Fox Terrier^13^	n.d			right abdominal^e^		
42	German Shepherd	7	L	n.d	n.d	MS	1
43		8			2nd left	MC	
44				intact	n.d	BN	
45		9			3rd left 5th left		2
46		10^*^			level of prepuce^e^		1
47		11*			right of prepuce^e^		
48		12		n.d	n.d	MC	
49		15			3rd left		
50	Golden Retriever	9*		neutered	n.d	BN	1
51		10		n.d			many
52	Labrador Retriever	2*		intact	4th left		1
53		2.5		n.d	4th^e^	MC	
54		7			1st^e^	BN	
55		7–13^ g^			n.d		
56							
57	Malamute	13*	L	neutered	4th left		
58	Maltese	7*	S	intact	n.d	H/D	
59		11*		neutered			many
60		12			5th left	MC	1
61	Pekingese	5		intact	4th right	H/D	
62	Rottweiler		L	n.d		BN	
63		7–13^ g^		n.d	n.d		
64		9		intact	5th left		
65					n.d		
66				n.d	2nd right	MC	
67	Shih Tzu	5*	S	neutered	5th left	BN	1
68		10*		intact	4th left 5th left		2
69	Spitz^13^	8*	M		right of prepuce ^e^		1
70	Springer Spaniel	9*		neutered	4th right	BN	
71		11*			4th left		
72	Toy poodle	10*	S		n.d	H/D	4
73	West Highland White Terrier	10*			level of prepuce ^e^	BN	1
No	Size of MGT [cm]	Predominant pattern	Histopathological evaluation, malignancy degree ^b^	Treatment	IHC markers MGT ^c^	Accompanying TTs and/ or cryptorchidism	References
1	n.d	n.d	simple carcinoma GIII	unilateral right mastectomy	n.d	no	(Figueiroa et al. 2012)
2			squamous cell carcinoma	surgical excision^f^		n.d	(Thakur et al. 2021)
3	0.5–3.0	papillary	simple adenoma		CNN, p63- pos	no	(Bearss et al. 2012)
4	1.0	n.d	complex adenoma	lumpectomy	ER-IR-60% PR IR-40%	n.d	(Saba et al. 2007)
5	2 × 2, 6 × 5		adenoma ^i^	surgical excision^f^	n.d	bilateral cryptorchid, Sertoli-cell tumour, seminoma	(Walker 1968)
6	0.5—3.0	acinar	simple adenoma		CNN, p63- pos	no	(Bearss et al. 2012)
7	7.5	papillary	fibroadenoma		different markers ^j^		(Maiti et al. 2014)
8	0.5–3.0	A. acinar B. papillary C. acinar/ papillary D. papillary	simple adenoma		CNN, p63- pos		(Bearss et al. 2012)
9		acinar					
10							
11							
12	1.5	acinar/ papillary	complex adenoma	simple mastectomy	no		(Han et al. 2016)
13	0.5–3.0	papillary	simple adenoma	n.d	CNN, p63- pos		(Bearss et al. 2012)
14	14, 1.5 2	n.d	adenocarcinoma GIII, invasive carcinoma in a benign mixed tumour	-unilateral right mastectomy -mastectomy and castration	ER, PR-pos	Leydig cell adenoma	(Kwon et al. 2017)
15	0.5–3.0	A. acinar/ papillary B. acinar C. papillary D. acinar	A., B., C.—simple adenoma D.-complex adenoma	surgical excision^f^ radiotherapy,	CNN, p63- pos	no	(Bearss et al. 2012)
16	6 × 4x3.5	papillary	cystadenocarcinoma		n.d		(Jabara 1969)
17	1.5	n.d	complex adenoma	lumpectomy	ER-IR-100% PR IR-60%	n.d	(Saba et al. 2007)
18	1		simple adenoma		ER-IR-60% PR IR-50%		
19	2		papillary cystadenoma, with transformation to squamous cell carcinoma and lobular hyperplasia (adenosis)	regional mastectomy	ER-IR-85% PR IR-50%		
20	1–2	n.d	tubulopapillary carcinoma, GI	surgical excision^f^	n.d	n.d	(Tkaczyk-Wlizło et al. 2023)
21	9	acinar	complex adenoma		different markers ^j^	no	(Maiti et al. 2014)
22	n.d	n.d	inflammatory carcinoma	n.d	COX-2, e-cadherin- pos	n.d	(Silva et al. 2019)
23	10		tubular carcinoma simple type, GI	surgical excision^f^	COX2, VEGFR2- no, ERα, ERβ, EGFR2-pos	no	(Arias et al. 2015)
24	6	papillary	simple adenoma		different markers ^j^		(Maiti et al. 2014)
25	n.d	acinar	carcinosarcoma		HER2, ER, PR-neg;CK5/6, VIM-pos		(Gopal et al. 2022)
26		n.d	tubulo-papillary carcinoma	n.d	ER-20% PR-60% EGFR-pos	n.d	(Carvalho et al. 2011)
27	7 × 5 × 4	papillary	adenocarcinoma	surgical excision during necropsy^k^	HER2, COX2, PCNA, Ki67-pos	no	(Saranya et al. 2022)
28	4 × 6x2	n.d	carcinoma in situ	-regional mastectomy; -new tumour, euthanasia	n.d	left: seminoma, right: Leydig cell tumour	(Zuchi et al. 2018)
29	n.d		intraductal carcinoma GI	n.d		n.d	(Di Giacomo et al. 2022)
30			tubulo-papillary carcinoma GII				
31	2		lipid-rich carcinoma, GII	n.d	CK14,VIM-pos, CK19, CK5/6, p63, CNN, ER, PR-neg	Leydig cell tumour	(Muscatello et al. 2021)
32	9 × 7x6		carcinoma–mixed type	surgical excision during necropsy^k^	n.d	no	(Jabara 1969)
33	0.5–3.0	acinar	simple adenoma	surgical excision^f^	CNN, p63- pos		(Bearss et al. 2012)
34	0.5	n.d	lobular hyperplasia (adenosis)	n.d	different markers^l^		(Schulman et al. 2022)
35	0.5–3.0	papillary	simple adenoma	surgical excision^f^	CNN, p63- pos		(Bearss et al. 2012)
36	8	acinar		surgical excision^f^	different markers ^j^		(Maiti et al. 2014)
37	13 × 10	n.d	basaloid adenoma		n.d		(Dąbrowski et al. 2011)
38	> 10		complex adenoma, GI				(Tkaczyk-Wlizło et al. 2023)
39	0.5–3.0	acinar	simple adenoma		CNN, p63- pos		(Bearss et al. 2012)
40	1	n.d	complex adenoma	lumpectomy	ER-IR-30% PR IR-5%		(Saba et al. 2007)
41	4 × 2.5		fibroadenoma	surgical excision^f^	n.d		(Jackson 1936)
42	n.d	n.d	osteosarcoma	n.d	n.d	n.d	(Slaska et al. 2016)
43			adenocarcinoma	surgical excision^f^			(Manjunatha et al. 2013)
44			fibroadenoma	simple mastectomy	ERα, p53, HER2 – neg; Ki-67,PR-pos;	no	(Mamom et al. 2012)
45	2	papillary	papillary cystadenoma	surgical excision^f^	n.d	n.d	(Veena et al. 2012)
46	9		simple adenoma		different markers^j^		(Maiti et al. 2014)
47	7	acinar					
48	n.d	n.d	carcinoma complex GI	n.d	n.d		(Slaska et al. 2016)
49	5 × 4 × 3.8		carcinoma in a mixed tumour GII	surgical excision during necropsy^k^	GATA3-pos; ER, PR,HER2—neg,	Leydig cell tumour, seminoma	(Machado et al. 2020)
50	0.5–3.0	acinar	simple adenoma	surgical excision^f^	CNN, p63- pos	no	(Bearss et al. 2012)
51	< 1	n.d	complex adenoma		n.d	n.d	(Tkaczyk-Wlizło et al. 2023)
52	0.5–3.0	acinar	simple adenoma		CNN, p63- pos	no	(Bearss et al. 2012)
53	15	n.d	carcinosarcoma	n.d	ER, p53-neg, CK14,PCK26-pos	n.d	(Lather et al. 2017)
54	n.d		lipoma		ER,p53-neg		
55	0.8		benign mixed mammary tumour	lumpectomy	ER-IR-40% PR IR-5%		(Saba et al. 2007)
56	3		complex adenoma		ER-IR-60% PR IR-50%	n.d	
57	0.5–3.0	acinar	simple adenoma	surgical excision^f^	CNN, p63- pos	no	(Bearss et al. 2012)
58	0.8	n.d	lobular hyperplasia (adenosis)	n.d	different markers^l^		(Schulman et al. 2022)
59	0.7						
60	small*	acinar	simple carcinoma ^m^	simple mastectomy	n.d	n.d	(Park et al. 2019)
61	1.5	n.d	lobular hyperplasia (adenosis)	surgical excision during necropsy^k^		cryptorchidism, Sertoli cell tumour	(Warland et al. 2011)
62	7 × 6.5x4		tubulo-papillary carcinoma, GII	unilateral mastectomy (inguinal lymph nodes removed)	GATA3, CK5/6-pos; ER, PR, HER2—neg	Sertoli cell tumour	(Machado et al. 2020)
63	2.5		benign mixed mammary tumour	lumpectomy	ER-IR-75% PR IR-40%	n.d	(Saba et al. 2007)
64	3.5	papillary	invasive papillary carcinoma	simple mastectomy	CK7,CK8/18-pos CK20 ~ IR; S100, Enolase-neg	no	(Ramírez et al. 2011)
65	n.d	n.d	simple adenoma		ERα, p53, HER2 – neg; Ki-67,PR-pos;		(Mamom et al. 2012)
66	5 × 4x3	papillary	anaplastic mammary carcinoma		n.d		(Aslan et al. 2017)
67	1.5	acinar/ papillary	complex adenoma	simple mastectomy	n.d	cryptorchidism	(Han et al. 2016)
68	2 3	acinar	benign mixed tumour			no	
69	5		simple adenoma	surgical excision^f^	different markers^j^		(Maiti et al. 2014)
70	0.5—3.0				CNN, p63- pos		(Bearss et al. 2012)
71		papillary					
72	0.1	n.d	lobular hyperplasia (adenosis)	n.d	different markers^l^		(Schulman et al. 2022)
73	0.5–3.0	acinar	simple adenoma	surgical excision^f^	CNN, p63- pos		(Bearss et al. 2012)
74							

^a^ size of the dog according to the American Kennel Club breed standards; viz. (S) Small (< 9 kg), (M) Medium (9,5–22,5 kg), (L) Large (23–45 kg), (G) Giant (> 45 kg); ^b^ tumour type based on guidelines published by Goldschmidt et al. (2011): acronyms: BN-benign neoplasms, H/D-hyperplasia, dysplasia, MC-malignant carcinoma, MM-malignant mixed mammary tumour, MS – malignant sarcoma;

^c^ immunohistochemical (IHC) markers: CNN – calponin; COX2—cyclooxygenase-2, e-cadherin; EGFR-;HER2- human epidermal growth factor receptor 2; ER—oestrogen receptor, GATA3—GATA binding protein 3; Ki-67- Ki-67 protein; p53- p53 protein, p63- p63 protein; PR—progesterone receptor; VIM-vimentin; IR- immunoreactivity; pos. -; positive immunoreactivity; neg. -; negative immunoreactivity

^d^ n.d.—no data; ^e^ not precisely indicated which mammary gland was affected by MGT; ^f^ types of surgical excision not indicated;^g^ 7–13 – only age range from 7 to 13 was indicated (Saba et al. 2007); ^h^ the dog was obese; ^i^ types of adenoma not described; ^j^ p53 (4/7),COX-2 (5/7), MMP7 – high—not indicated in which dog (Maiti et al. 2014); ^k^ euthanasia was needed due to the poor condition of the dog, MGT was collected during necropsy; ^l^ different markers including: CNN, CK5/6, CK7, CK14, CK19, p63, VIM but not indicated in which dog these markers were IR (Schulman et al. 2022); ^m^ simple carcinoma was assessed based on mammary gland cytology, the owner refused to perform histopathological examination (Park et al. 2019)

^*^no history of obesity, diabetes, or sex hormonal therapy

### Incidence of male MGTs

For many years, the incidence of MGTs in male dogs was evaluated in the literature as low, i.e. 0.5–2.7%, usually < 1% (Bearss et al. 2012; Lather et al. 2017). However, the available data indicate that, depending on the country and the size of the tested group, the incidence of male MGTs varies from 0.5% (4/341) in Croatia (Šoštarić-Zuckermann et al. 2013), 1.7% (6/357) in Canada (Mitchell et al. 1974), 2.2% (25/1142) in Italy (Merlo et al. 2008), 2.5% (2/79) in South Korea (Chae et al. 2007), and 2.6% in China (13/504)(Zheng et al. 2022) to 5% (3/63) in India (Dhami et al. 2010; Lather et al. 2017) and Poland (5/92) (Tkaczyk-Wlizło et al. 2023).

### Histopathological types of MGTs in male dogs

**Fig. 1:**
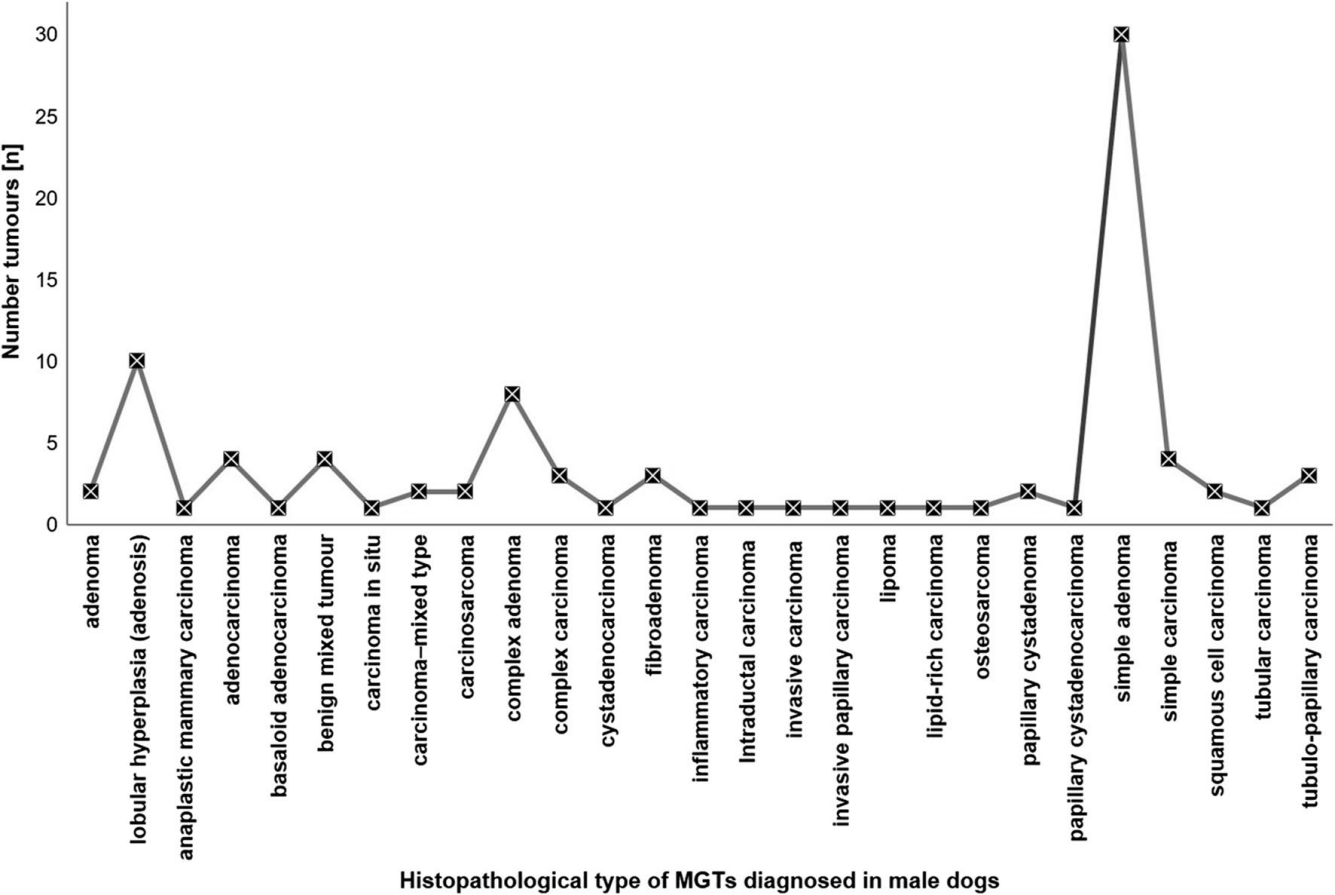
Incidence of different histopathological types of MGTs in male dogs

The literature review showed that 92 mammary gland tumours were diagnosed in 74 male dogs. The majority of collected samples were benign neoplasms (BNs, 54.3%). Histopathologically, most of BNs were simple adenomas (34.8%) (Bearss et al. 2012; Maiti et al. 2014; Saba et al. 2007) and complex adenomas (8.7%) (Bearss et al. 2012; Saba et al. 2007). The other non-malignant tumours were identified as benign mixed tumours (4.3%) (Han et al. 2016; Saba et al. 2007), fibroadenomas (3.3%) (Jackson 1936; Maiti et al. 2014), and papillary cystadenomas (2.2%) (Saba et al. 2007; Veena et al. 2012) (Table 1, Fig. 1).

As above-mentioned, simple adenomas are the most commonly occurring neoplasms in male dogs; this is also confirmed by other scientific groups (Bearss et al. 2012; Maiti et al. 2014). To date, there is only one paper describing the largest group of male dogs with MGTs. Bearss et al. (2012) analysed 18 male dogs with 27 MGTs; 26 neoplasms (96.3%) were simple adenomas and one tumour was a complex adenoma. In another paper, five out of seven (71.4%) affected male dogs had simple adenomas, one complex carcinoma and one papillary fibroadenoma (Maiti et al. 2014).

Histopathologically, a simple adenoma is a benign neoplasm characterised by a well-demarcated nodular lesion composed of numerous acinar and papillary structures and lined by a single layer of cuboidal to columnar epithelium. The neoplastic cells are supported by a scant to moderate fibrovascular stroma (Goldschmidt et al. 2011; Maiti et al. 2014). The round to oval nuclei are located centrally, the chromatin is finely stippled, and a small central nucleolus is observed (Goldschmidt et al. 2011).

Microscopic evaluations indicate a predominant acinar or papillary histological pattern in simple adenomas. Currently, the majority of described adenoma-type neoplasms are mainly characterised by an acinar pattern (Bearss et al. 2012; Maiti et al. 2014). In this study, the predominant histological pattern was not indicated in each case. Nevertheless, our analysis based on available data showed co-occurrence of both histological patterns of adenomas (Table 1).

Other reported MGTs were malignant neoplasms mainly of epithelial origin (28.3%) such as adenocarcinomas (5.4%) (Kwon et al. 2017; Manjunatha et al. 2013; Saranya et al. 2022), simple carcinomas (4.3%) (Figueiroa et al. 2012; Park et al. 2019), and tubulo-papillary carcinomas (3.3%) (Carvalho et al. 2011; Di Giacomo et al. 2022; Machado et al. 2020) or in the mixed type: carcinosarcoma (2.2%) (Lather et al. 2017). Also individual cases of other types of carcinomas, e.g. carcinoma in situ (Zuchi et al. 2018), inflammatory carcinoma (Silva et al. 2019), intraductal papillary carcinoma (Di Giacomo et al. 2022), or complex carcinoma (Slaska et al. 2016), were reported. Moreover, a rare case of a male malignant tumour of mesenchymal origin (osteosarcoma) was described by Slaska et al. (2016).

Ten out of the 92 (10.9%) diagnosed tumours in male dogs were non-neoplastic mammary alterations, i.e. lobular hyperplasia (adenosis) (Table 1) (Schulman et al. 2022; Warland et al. 2011). The first study described four males with seven teat sinus and duct adenomatous hyperplasias of mammary glands (Schulman et al. 2022). On the other hand, Saba et al. (2007) described a dog with a malignant papillary cystadenoma with transformation to squamous cell carcinoma, and peripheral mammary lobular hyperplasia was noted as well.

### Number, size, and localisation of MGTs

Fourteen (18.9%) of the described canine cases of male MGTs had more than one neoplasm; the ratio of these dogs to individuals with only one neoplasm was approximately 1:4. Half of these tumours were two masses, other cases included three (7.2%), four (21.4%) or more numerous neoplasms (21.4%). The highest number of determined mammary abnormalities, i.e. four, in form of adenomas were detected in 2 dogs (Bearss et al. 2012) and four adenomatous hyperplasias were found in one case (Schulman et al. 2022). The first paper described one dog which had two masses on the left of the prepuce and two additional masses on the right; all were simple adenomas. The last case, a male Cocker Spaniel, had one neoplasm in each mammary gland: 1st left and right, 2nd left, and 3rd left. Three of the four neoplasms were simple adenomas and the last one (1st right) was a complex adenoma (Bearss et al. 2012) (Table 1).

Due to the rarity of male MGTs and probable owner’s unawareness of the possibility of development of MGTs in male dogs, mammary abnormalities are diagnosed late. Based on the WHO classification (Owen and World Health Organization 1980), the minority of diagnosed neoplasms were small (up to 3 cm)(Bearss et al. 2012; Han et al. 2016; Saba et al. 2007), but others were definitely large tumours (> 5 cm) from 6 (Jabara 1969; Maiti et al. 2014; Walker 1968; Zuchi et al. 2018) to 13–15 cm (Dąbrowski et al. 2011; Kwon et al. 2017; Lather et al. 2017).

The majority of the described MGTs were found in the last two pairs: caudal abdominal and inguinal mammary glands. Some authors did not indicate which of the last two pairs were affected and only the “level of prepuce” annotation is available. Information of the tumour localisation was available in 74 out of the 92 MGTs; 43 (58.1%) neoplasms developed in 4th or 5th canine mammary glands (Table 1). This is consistent with reports from other authors who observed that most MGTs affected the posterior mammary glands^.^(Jabara 1969; Maiti et al. 2014; Pinello et al. 2022). This may be supported by the fact that mass of these mammary glands is greater than that of the other glands, which is associated with increased susceptibility to injury or carcinogenic stimuli attributable to the more pendulous nature of these glands. This may result in a higher frequency of neoplastic lesions in this region of the mammary ridge (Bearss et al. 2012; Han et al. 2016; Maiti et al. 2014).

The number, size, and localisation of the neoplasms were not related to the histopathological type of the diagnosed MGTs.

### Treatment and procedures

**Fig. 2:**
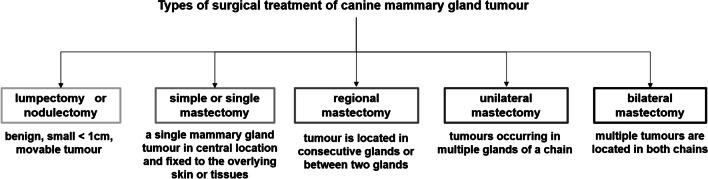
Types of surgical tumour removal performed in canine tumour treatment based on Papazoglou et al. (2014)

Surgical excision is the gold standard of veterinary treatment of mammary tumours in dogs (Kwon et al. 2017; Papazoglou et al. 2014). Depending on the number of tumours, their size, and occupation of lymph nodes, the surgery may include only the affected teat via lumpectomy (Saba et al. 2007) or regional mastectomy (Manjunatha et al. 2013; Zuchi et al. 2018), but the largest or more numerous neoplasms are removed during unilateral (Kwon et al. 2017; Machado et al. 2020) or bilateral mastectomy (Papazoglou et al. 2014) (Table 1, Fig. 2).

Other treatments, such as radiotherapy, were applied in some cases; however, due to its continuous growth, the neoplasm was eventually removed surgically (Jabara 1969). In another case, the MGT was a triple-negative neoplasm, hence chemotherapy was proposed but the owner did not agree (Machado et al. 2020). Furthermore, there are examples of older animals that suffered badly from MGTs and accompanying diseases; thus, it turned out at the time of the appointment at the veterinary clinic that they had to be euthanised (Machado et al. 2020; Saranya et al. 2022).

### Characteristics of individuals

Tumorigenesis is an unwanted phenomenon in female and male dogs. Therefore, the characteristics of individual animals are analysed to identify factors related to a higher probability of MGT development, i.e. the age, breed of dog, or hormonal exposure. The recognition of their impact may help to prevent or recognise the disease at an appropriate time.

#### Age, breed, and size of the animal

**Fig. 3:**
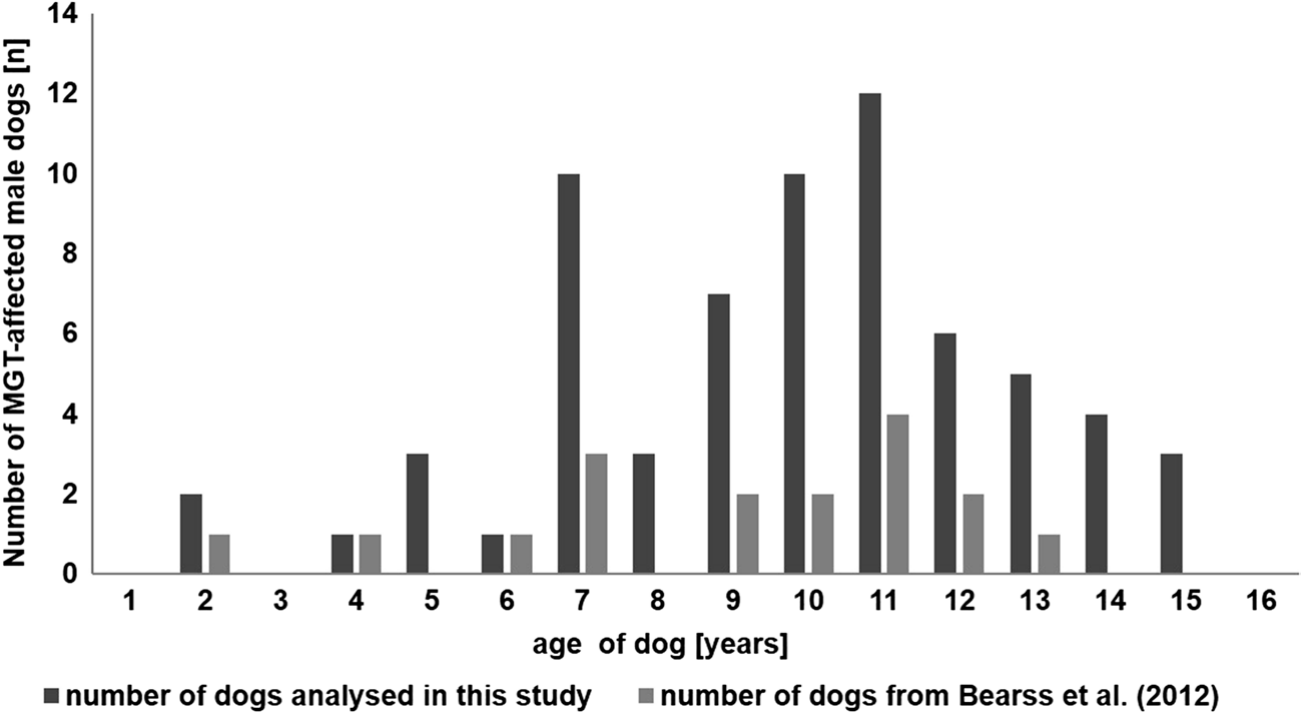
Age-associated incidence of MGTs in male dogs collated with results obtained by Bearss et al. (2012)

One of the important factors in the aetiology of the disease is the age of the patient. In our study, the age of the male dogs varied from 2 to 15 years, but an increased number of male MGTs were observed at the age from 7 to 13 years with an age peak at 11 years (Fig. 3). So far, only one paper presented the highest number of male cases of MGTs, i.e. 18 dogs with 27 MGTs described by Bearss et al. (2012). Therefore, we compared our results with the data obtained by Bearss et al. (2012) (Fig. 3). Interestingly, two age peaks were observed: 7 years and an increased incidence of MGTs between 10–12 years with special emphasis on 11 years. It should be noted that the age of the dog was not connected with its breed (Table 1).

It is interesting that among the described cases of male MGTs, 15 out of 74 (20.2%) were diagnosed in Crossbreeds (Arias et al. 2015; Gopal et al. 2022; Maiti et al. 2014; Silva et al. 2019) followed by Cocker Spaniels (18.9%) (Bearss et al. 2012; Han et al. 2016; Kwon et al. 2017; Maiti et al. 2014), German Shepherds (10.8%) (Machado et al. 2020; Maiti et al. 2014; Veena et al. 2012), Labrador Retrievers (6.7%) (Bearss et al. 2012; Lather et al. 2017; Saba et al. 2007) and Rottweilers (6.7%) (Machado et al. 2020; Mamom et al. 2012; Ramírez et al. 2011; Saba et al. 2007). Moreover, previous literature data indicated that Cocker Spaniels may be overrepresented among male dogs with MGTs (Bearss et al. 2012). In the case of the other breeds, only rare occurrence of MGT was noted in e.g. Akita (Figueiroa et al. 2012), Boxer (Walker 1968), and Dachshund (Bearss et al. 2012).

The analysis of the body weight of the affected animals in our study showed that generally MGTs did not occur in giant dogs (> 45 kg). In turn, neoplasms were diagnosed in small dog breeds, e.g. English Bulldog, Fox Terrier, Maltese, Shih Tzu, and West Highland White Terriers. The majority of the MGT-affected purebred dogs were medium (especially Cocker Spaniels) and large (such as German Shepherds, Labrador Retrievers) dogs (Table 1).

#### Sexual status, IHC markers, and additional factors

A study performed on intact female dogs showed that they were at higher risk of MGT development than neutered animals. Thus, ovariohysterectomy has a protective effect if it is performed before the second oestrus at the latest (Schneider et al. 1969). Currently, the role of sex hormones and castration in male dogs and their association with the occurrence of mammary neoplasia is still unknown (Mamom et al. 2012; Saba et al. 2007; Silva et al. 2019). To date, no study has been conducted to verify the thesis that castration is associated with a lower risk of MGTs in male dogs.

**Fig. 4:**
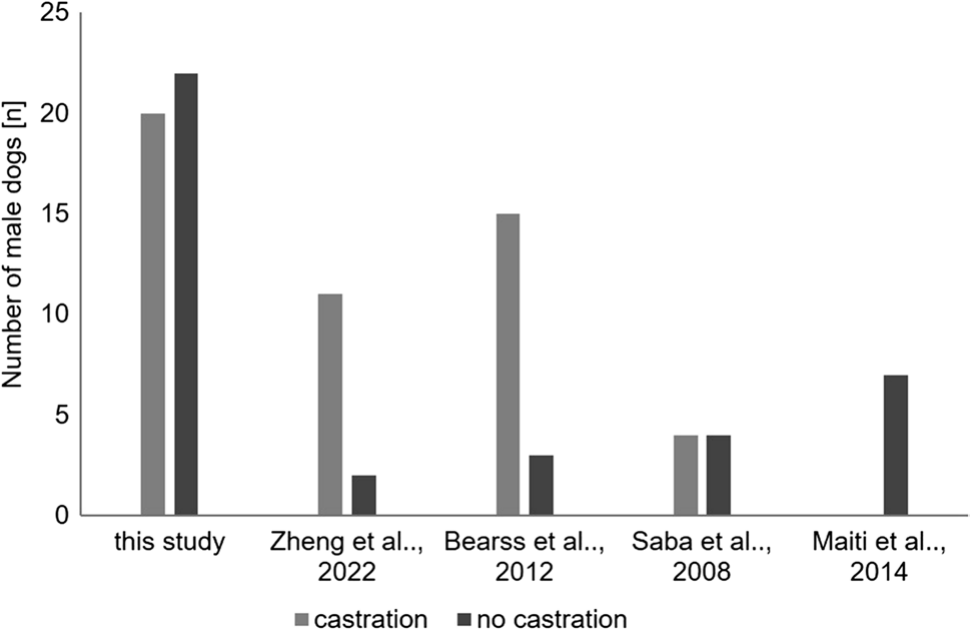
Impact of the sexual status (C-castration, NC- no castration) of male dogs on development of MGTs based on results presented in this study and obtained by other scientific groups

Based on reports where information of the sexual status of the dog was indicated, the number of intact and neutered male dogs in this study was almost equal (52.4% to 47.6%, 1:1) (Table 1, Fig. 4). On the other hand, data obtained by other scientific groups showed inconsistent results due to the limited number of tested dogs, hence no conclusions can be drawn at present. Therefore, it is necessary to collect and analyse male MGTs to obtain more data required for discussion of the possible impact of the sexual status on the development of MGTs in male dogs. Also, the most appropriate time of castration should be assessed, as in the case of females.

Despite the lack of data confirming or negating the impact of castration on tumour development, there is evidence linking sexual hormones such as oestrogen (ER) and progesterone (PR) with mammary carcinogenesis (Canadas-Sousa et al. 2019). Tests based on ER and PR immunoreactivity and other immunohistochemical (IHC) factors, including such proteins as p53, Ki-67, and Her-2/neu, have been applied in the diagnosis of female mammary neoplasms for years. However, still little is known about the expression of different IHC markers in male dogs with MGTs (Mamom et al. 2012).

The limited knowledge about male MGTs prompted the application of many different IHC markers, including hormone receptors: oestrogen receptor alpha, progesterone receptor, such cytokeratins as 5/6, 7, 8/18, 15 and proteins p53, p63, calponin, and GATA3 to gain new knowledge of the tumour behaviour (Table 1). Saba et al. (2007) confirmed a strong impact of sexual hormones on MGTs in eight male dogs (intact and neutered). Six of the eight dogs had strong ER immunoreactivity in 50% of the neoplastic cells, and expression of PR was observed in 50% of the neoplastic cells in seven dogs. However, not in each case was the positive immunoreactivity to sexual hormones observed. Machado et al. (2020) reported two cases of male mammary carcinoma (no data about the sexual status of these animals) which were triple-negative for such markers as ER, PR, and HER2. The application of basal cytokeratins markers allowed differentiating the neoplasms into basal-like and non-basal subtypes. Human medicine literature shows that triple-negative mammary neoplasms in males often have a poor prognosis due to their aggressive behaviour. Also, the described male dogs (carcinoma in a mixed tumour GII, tubulo-papillary carcinoma GII) died soon; the first dog passed away 6 months after the surgery and the second one was euthanised at the time of the diagnosis. Mamom et al. (2012) reported two non-castrated dogs, one with a simple adenoma and another with a diagnosed fibroadenoma. Immunohistochemical tests showed that both tumours were strongly positive for PR, negative for ER, Her-2/neu, and p53, and slightly immunoreactive to Ki-67. The authors speculated that the absence of ER may have resulted from the impact of androgen hormones, but no report on the correlation of sex hormones and the occurrence of MGTs in male dogs is available Mamom et al. (2012). The above-mentioned data indicate variability and complexity of the biology of tumour behaviour. Therefore, the application of IHC markers is an important step in elucidation of the development and growth of tumours.

Other factors, such as obesity, diabetes, and application of sex hormonal therapy, which may have an impact on hormonal imbalance, were excluded in 39.1% of MGT-affected dogs (Bearss et al. 2012; Han et al. 2016; Silva et al. 2019). Interestingly, Walker (1968) described a case of an extremely fat Boxer (43 kg; the average is 30 kg) diagnosed with two MGTs and, additionally, a Sertoli-cell tumour in the right testicle and a seminoma in the left. However, the data on obese dogs are limited only to a few cases (Gopal et al. 2022; Walker 1968); hence, it is not possible to directly relate obesity with tumorigenesis.

### Testicular tumours

Testicular tumours (TTs) represent more than 90% of neoplasms of the canine male genitalia (Hohšteter et al. 2014). Among TTs, seminomas (SEM) derived from germ cells and neoplasms originating from sex-cord stroma i.e. Sertoli cell tumours (sertoliomas; SCT) and (interstitial) Leydig cell tumours (leydigomas; LCT) are the most commonly diagnosed in dogs and pose up to 98% of canine cases (Hohšteter et al. 2014; Manuali et al. 2020; Nascimento et al. 2020).

The above-mentioned tumours may develop in normal testicles, but it has been noticed that cryptorchidism is a predisposition factor for testicular tumour development. The available data indicate a 20-fold higher incidence of SCT in cryptorchid than scrotal testes (Choi et al. 2008). However, a study performed by Nascimento et al. (2020) showed that seminoma (34.9%) was the most frequent tumour among cryptorchid dogs followed by SCT (30.2%).

SCT arises from Sertoli cells of seminiferous tubules. The intersection of the tumour usually displays a firm, whitish, well-demarcated neoplasm within the testicular parenchyma. Histopathologically, SCT cells are arranged in islands or tubules supported by abundant fibrous tissue; they are classified as intratubular or diffuse SCTs (Manuali et al. 2020). Another type of TTs, i.e. interstitial cell tumours also called LCTs, often protrude from the testicle and histopathologically are classified into cystic-vascular, solid-diffuse, and pseudoadenomatous. In turn, SEMs are classified into the intratubular type (early stage of tumour development) and the diffuse type (large nodular aggregates) (Manuali et al. 2020).

A pathogenic association between oestrogen production and development of TTs has been observed (Walker 1968). One in five dogs with SCTs displays signs of feminisation syndrome (FS) resulting from hyperoestrogenism (Gopinath et al. 2009). The association of FS with TTs was prompted by the observation that signs of feminisation in male dogs disappear when the tumour is removed and reoccur when functional metastases are present (Gopinath et al. 2009). The signs of feminisation include alopecia, attractiveness to other male dogs, pendulous prepuce, and mammary gland abnormalities, e.g. gynaecomastia, enlarged nipples and glands, mastitis, mammary hyperplasia, or benign mammary gland tumours (Choi et al. 2008; Walker 1968; Warland et al. 2011).

### Relation between MGTs and TTs

Factors resulting in hormonal imbalance assigned to development of mammary neoplasms in males are suggested as predisposing factors for testicular tumours (TTs) and vice versa (Machado et al. 2020). For many years, it has been suggested that the development of MGTs may also be related to tumorigenesis in testicles (Maiti et al. 2014; Walker 1968). As shown by the analysis of available papers, no TTs were reported in 48 of 74 male dogs. Only in seven cases (12.7%), TTs were diagnosed concurrently with MGTs or the disease was diagnosed in past or after MGTs (Table 1). Additionally, in three cases, cryptorchidism was diagnosed, which is a serious factor for development of TT in future (Han et al. 2016; Walker 1968; Warland et al. 2011).

## Conclusions

Although MGTs in male dogs are not as common as in female dogs, they should be taken into consideration in the differential diagnosis of subcutaneous mass, especially around the caudal abdominal and inguinal mammary glands and the prepuce in male dogs (Han et al. 2016). It is important to perform palpation of the mammary chains in all dogs, regardless of their sex (Saba et al. 2007).

Currently, a gold standard in the recognition of canine MGT is a histopathological evaluation, which allows assessment of the type of tumour and the degree of malignancy (Goldschmidt et al. 2011). The application of different immunohistochemical markers additionally helps to determine factors of the tumour behaviour, e.g. sexual hormone receptors or proteins associated with tumorigenesis. A study performed by Saba et al. (2007) confirmed the immunoreactivity to oestrogen and progesterone receptors in the majority of tested samples; the role of sex hormones may be crucial in the development and growth of male MGTs. In turn, Machado et al. (2020) and Gopal et al. (2022) reported cases of triple-negative carcinomas.

The presented results showed that the majority of male MGTs were benign neoplasms, with special reference to the development of adenomas (simple, complex) followed by adenomatous hyperplasia (Table 1, Fig. 1). It is interesting that this type of mammary gland abnormalities occurs frequently in male dogs. The incidence of malignant MGTs in male dogs is significantly lower than in female dogs. In our study, up to four cases of a special type of malignant carcinomas were noted.

Based on available data, only 12.7% of MGTs were associated with the development of TTs. Therefore, no general association between these tumours should be assumed. However, consideration should be given to the benign mammary gland abnormalities in male dogs resulting from oestrogenic activity (feminisation syndrome) of testicular tumours (Walker 1968; Warland et al. 2011) and examples of MGTs and TTs occurred simultaneously.

Due to limited data on male MGTs, it is currently not possible to indicate a potential protective effect of castration or the impact of the excess weight. Therefore, the authors encourage other scientific groups to publish as many details of tumour behaviour and characteristics of individuals as possible. Each new case of MGT in a male dog is important for further studies.

## Data Availability

The authors confirm that the data supporting the findings of this study are available within the article.
